# Effect of low-dose naltrexone for long COVID: a systematic review and meta-analysis

**DOI:** 10.1136/bmjopen-2025-111253

**Published:** 2026-07-16

**Authors:** Oyungerel Byambasuren, Tiffany Atkins, Shaira Baptista, Paul Glasziou, Samantha Chakraborty

**Affiliations:** 1 Institute for Evidence-Based Healthcare, Bond University, Robina, Queensland, Australia; 2 Australian Living Evidence Collaboration, School of Public Health and Preventive Medicine, Monash University, Melbourne, Victoria, Australia

**Keywords:** COVID-19, Medicine, Systematic Review

## Abstract

**Abstract:**

**Objective:**

Long covid is a debilitating chronic condition, and the effect of low-dose naltrexone (LDN) on its symptoms is unclear. We aimed to determine the effectiveness of LDN on symptoms of long covid.

**Design:**

Systematic review and meta-analysis.

**Data sources:**

PubMed, Embase and Cochrane Library for published studies; ClinicalTrials.gov and WHO International Clinical Trials Registry Platform (ICTRP) for registered ongoing studies were searched through 5 May 2026.

**Eligibility criteria:**

We included randomised controlled trials and pre–post studies of patients with long covid reporting on fatigue, quality of life, cognitive symptoms or function and other long covid symptoms.

**Data extraction and synthesis:**

Two independent reviewers used standardised methods to search, screen and select included studies. Risk of bias was assessed using the Newcastle-Ottawa Scale. Meta-analysis was conducted using random effects models.

**Results:**

Of 397 titles and abstracts screened, no randomised controlled trials were identified. Four observational pre–post studies from the USA and Ireland (n=155) met inclusion criteria. LDN doses varied from 1 mg/day to 6 mg/day. Pooled pre–post analyses showed moderate effects for reducing fatigue (Hedges’ g=−0.74; 95% CI −1.11 to −0.37; p<0.001), brain fog (Hedges’ g=−0.53; 95% CI −1.01 to −0.05; p=0.03) and improving sleep quality (Hedges’ g=−0.60; 95% CI −0.91 to −0.30; p=0.0001), and large effects for pain (Hedges’ g=−0.93; 95% CI −1.29 to −0.57; p<0.001) and daily functioning (Hedges’ g=−0.93; 95% CI −1.29 to −0.57; p<0.0001) in favour of LDN. Heterogeneity ranged from 0% to 62%. No serious adverse events were reported in the two studies that assessed safety.

**Conclusion:**

Limited evidence from small pre–post studies suggests LDN may improve fatigue, cognition, sleep, pain and functioning in long covid. However, certainty of evidence is low. Well-powered trials are needed to confirm efficacy, determine dosing and duration and identify subgroups most likely to benefit.

**Trial registration:**

https://doi.org/10.17605/OSF.IO/C2VKX

**STRENGTHS AND LIMITATIONS OF THIS STUDY**

- This systematic review includes an up-to-date, comprehensive search of multiple databases, capturing both published and unpublished studies on low-dose naltrexone for long covid.
- No published randomised controlled trials were identified, although several are ongoing.
- All included studies were uncontrolled pre–post designs, limiting causal inference.
- Observed improvements may reflect the natural course of illness, regression to the mean or placebo effects, particularly as outcomes were self-reported.

## Introduction

 The COVID-19 pandemic has given rise to a novel chronic condition known as post-acute sequelae of COVID-19 or long covid. This condition is characterised by persistent symptoms such as fatigue, respiratory issues and cognitive impairments, which can cause prolonged illness and disability.^1 2^ Conservative estimates suggest that around 10% of people infected with COVID-19 develop long covid, translating to millions of cases worldwide.^3^

Among the many symptoms of long covid, the most debilitating are fatigue, post-exertional malaise and cognitive dysfunction.^4^ These symptoms have profound personal, social, psychological and financial consequences.^5^ There is an urgent need for effective, evidence-based treatment options for long covid.

There is substantial symptom overlap between myalgic encephalomyelitis/chronic fatigue syndrome (ME/CFS) and long covid. Both conditions are also associated with impairment of the transient receptor potential cation channel subfamily M member 3 (TRPM3) ion channel, pointing to a potential common underlying biological mechanism and highlighting TRPM3 as a therapeutic target.^6 7^

One proposed off-label treatment for this impairment is low-dose naltrexone (LDN). Naltrexone in standard doses (50–100 mg/day) is approved as an opioid antagonist for treating alcohol use disorder and opioid dependence.^6 7^ In contrast, much lower doses (~4.5 mg/day) have been used to manage chronic pain, ME/CFS and other chronic neuroimmune disorders such as Crohn’s disease and fibromyalgia, due to its biological plausibility and reported tolerability.^7^,^10^

Although clinical effectiveness studies are scarce, off-label use of LDN is increasing.^11 12^ This review therefore aims to systematically summarise all available evidence on the effectiveness of LDN for alleviating symptoms of long covid.

## Methods

We conducted a systematic review using enhanced processes and automation tools and reported it according to the Preferred Reporting Items for Systematic Reviews and Meta-Analyses (PRISMA) statement^13^ and Meta-analysis of Observational Studies in Epidemiology (MOOSE) checklist.^14^ Our protocol was pre-registered on the Open Science Framework (OSF) (https://doi.org/10.17605/OSF.IO/C2VKX). Ethics approval was not required.

### Search strategy

We searched PROSPERO and OSF databases to confirm no similar reviews were underway. We then searched PubMed, Embase (Elsevier) and the Cochrane Library for published studies, ClinicalTrials.gov and WHO International Clinical Trials Registry Platform (ICTRP) for registered ongoing studies from inception to 5 May 2026.

Search strings combined Medical Subject Headings (MeSH) or other subject terms, synonyms and search filters, and were designed with the Systematic Review Accelerator suite of tools.^15^ An information specialist peer-reviewed the strategy using Peer Review of Electronic Search Strategies (PRESS) guidelines (see online supplemental 1). No restrictions were applied for language or publication type. We performed backward and forward citation searching using SpiderCite.^15^

### Eligibility criteria

We included randomised controlled trials (RCTs) and pre–post cohort studies enrolling patients with long covid, defined according to the WHO clinical case definition, where the majority of participants were at least 12 weeks post-infection.^16^ Case studies and case series were excluded. Studies conducted in either primary or secondary care settings were eligible.

- Participants: Adults with long covid (with the majority at least 12 weeks post-covid or with a reported subset) were eligible for this review. Studies focusing on acute and post-acute covid (within 12 weeks of the onset of COVID-19 symptoms) were excluded.
- Interventions: LDN (1–10 mg) as sole or add-on intervention.
- Comparator: usual care or placebo.
- Outcomes: The primary outcomes were fatigue and health-related quality of life. Secondary outcomes included cognitive symptoms or cognitive function, other long covid-related symptoms and adverse events (including gastrointestinal symptoms). Outcomes were included if they were measured using either validated patient-reported outcome measures or clearly defined symptom scales as reported by the original studies; no restrictions were placed on specific instruments a priori due to expected heterogeneity in outcome measurement across early-stage studies.
  Where studies reported outcomes using different validated scales measuring the same construct (eg, fatigue or quality of life), results were standardised using standardised mean differences (SMDs) to enable comparability across instruments. Extracted outcomes reflected post-intervention time points reported in each study, with no restriction on follow-up duration due to variability in study design and reporting in the available literature. All available post-treatment assessments were included as reported.
  No formal exclusion criteria were applied based on outcome measurement tool or follow-up duration, in order to capture the breadth of emerging evidence in this early field.
  Given the heterogeneity and early-stage nature of the evidence base, outcome definitions and measurement instruments were accepted as reported in the primary studies, with synthesis undertaken descriptively and through standardisation where appropriate.

### Study selection and screening

Two authors (OB and SC) independently screened titles and abstracts. Full texts of potentially eligible articles were retrieved and assessed by OB and SC. Citation and trial registry searches were screened by OB and TA. Disagreements were resolved by discussion or consultation with all authors.

### Data extraction

We piloted a standardised form on three studies and then extracted data in duplicate (TA and SB). Extracted items included:

- Study details: study authors, year, country, study design, duration of follow-up, setting.
- Participant characteristics: sample size, age, gender, time since COVID-19 infection.
- Intervention details: dose, frequency and comparator/s.
- Reported outcomes: fatigue, quality of life, cognitive symptoms or function, other COVID-19 symptoms, adverse events.

### Risk of bias assessment

We planned to use the Cochrane Risk of Bias 2.0 tool for RCTs and the Newcastle-Ottawa Scale (NOS) for observational studies.^17^ Two authors (TA and SB) independently assessed risk of bias. For pre–post designs, we adapted the NOS by removing non-applicable items and adjusting the scoring accordingly. Given the uncontrolled before–after design of included studies, the NOS was used to provide a structured descriptive assessment of methodological limitations.

### Data analysis

Effect sizes for pre–post changes were calculated using Hedges’ g (SMD with small-sample correction). Where effect sizes were not directly reported, they were derived from available summary statistics (eg, p values for within-group comparisons) using established methods, with approximations made where reporting was incomplete in the primary studies. Calculations were supported using the Campbell Collaboration effect size calculator^18^ in line with guidance from the Cochrane Handbook for Systematic Reviews of Interventions.^19^

Effect sizes were interpreted using conventional thresholds: negligible (<0.20), small (0.20–0.49), moderate (0.50–0.79) and large (≥0.80).^20^ Scale directions were harmonised prior to analysis so that negative values consistently represented symptom improvement or functional benefit favouring LDN. Where multiple measures of the same construct (eg, fatigue) were reported within a study, a single outcome was selected based on consistency with other included studies to enable pooling, ensuring that each study contributed only one effect size per outcome domain. Some study results were not included in the meta-analysis due to differences in outcome reporting or insufficient data to calculate effect sizes.

Pooled effect sizes were estimated using a random-effects model (DerSimonian-Laird), selected a priori to account for anticipated clinical and methodological heterogeneity across studies, irrespective of the magnitude of statistical heterogeneity (I²). Statistical heterogeneity was assessed using the I² statistic. Publication bias was not assessed due to the small number of included studies (<10). Planned subgroup analyses (eg, baseline severity, LDN dose) were not undertaken due to insufficient data.

All analyses were conducted using Stata V.16.1/MP (StataCorp, 2019).

Patient and public involvement in research: None.

## Results

**Figure 1:**
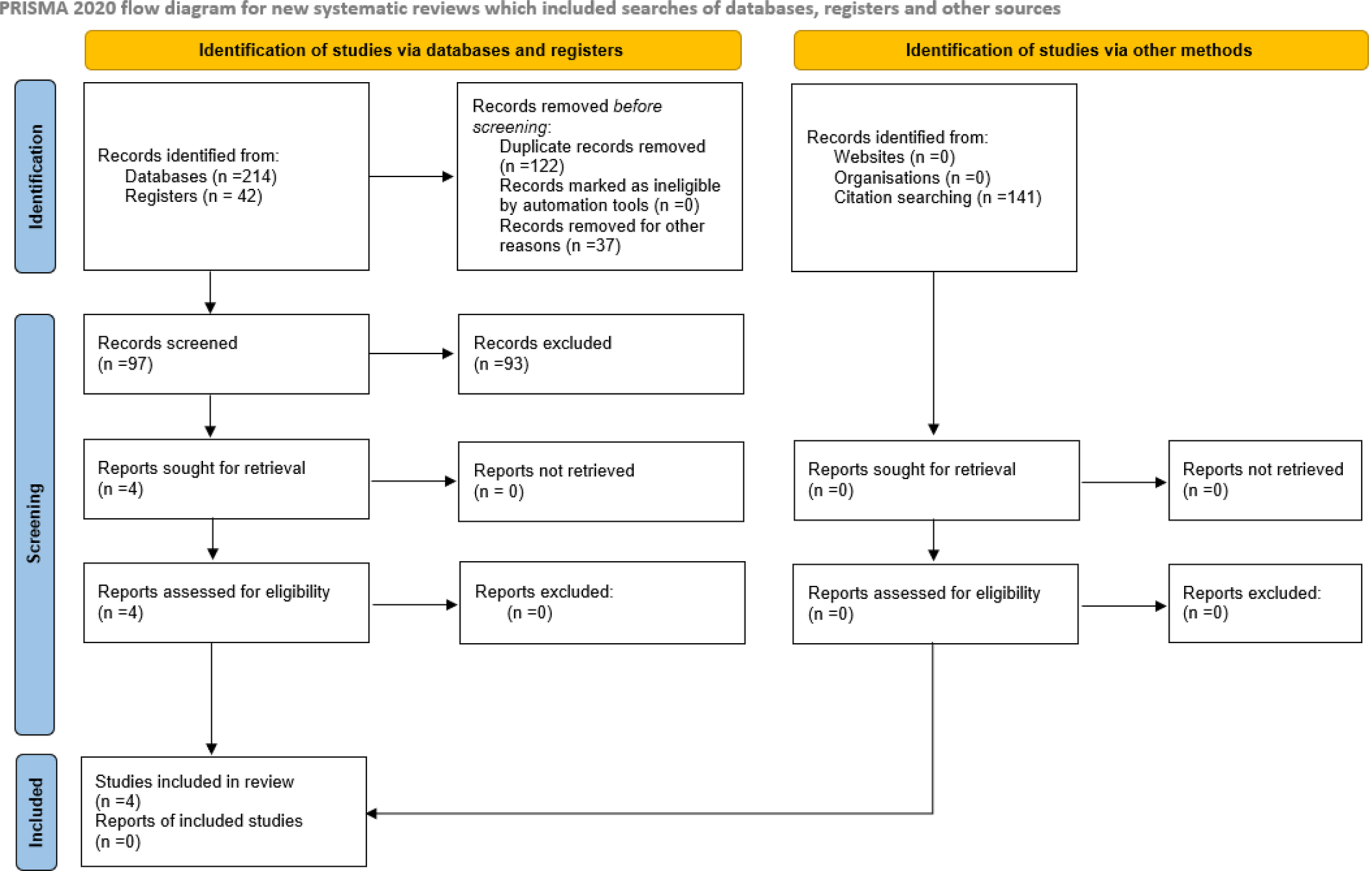
Screening and selection of studies. PRISMA, Preferred Reporting Items for Systematic Reviews and Meta-Analyses.

Of 397 records identified, 4 full-text articles met the inclusion criteria (figure 1). All included studies were pre–post cohort designs, conducted in the USA (n=3) and Ireland (n=1), and together included 155 patients^21^,^24^ (table 1). LDN was used as a standalone intervention in three studies; one study^22^ combined it with nicotinamide adenine dinucleotide. Two studies reported minor adverse events.^22 23^ No RCTs were identified, but four registered trials were ongoing at the time of this review with no published results available.

**Table 1:** Characteristics of included studies

Study ID, location	Study type, timeframe	Population	Intervention	Outcomes	Adverse events
Bonilla *et al*, USA^21^	Retrospective cohort, 18 May 2021 to 18 Mar 2023	N=59, median age 45, 68% female, median duration of symptoms 361 days	Individualised LDN dose-titration ranging from 0.5 mg/day to 6 mg/day for a median of 143 days	Total number of symptoms, severity in subset of symptoms (fatigue, PEM, unrefreshing sleep and abnormal sleep pattern using study questionnaire Likert scale) and FSS	No information provided
Isman *et al*, USA^22^	Prospective cohort, 31 Mar 2021 to 22 Dec 2022	N=36, mean age 45 (28–69), 70% female, mean duration of symptoms 223 days	LDN titrated from 1 mg/day for 4 days, 2 m/day for 4 days and 4.5 mg/day thereafter, 400 mg/mL NAD+ was applied via iontophoresis patches weekly for 12 weeks	Fatigue (Chalder Fatigue Scale), pain and quality of life (SF-36)	Mild adverse events (skin irritation at location of NAD+ patch, nausea, fatigue, dizziness and low mood)
O’Kelly *et al*, Ireland^23^	Prospective cohort, Jun to Nov 2020	N=36, median age 44 years, 77% female, median duration of symptoms 333 days.	LDN 1 mg/day in first month, 2 mg/day in second month, 3 mg/day for third month.	Recovery from COVID-19, limitation in activities of daily living, energy levels, pain levels, levels of concentration and sleep disturbance using study questionnaire with Likert scale	Two participants had same adverse events (diarrhoea and fatigue)
Tamariz *et al*, USA^24^	Retrospective cohort, 2021 to Jan 2023	N=24, mean age 54, 34% female, (duration of symptoms not reported)	LDN 1.5–4.5 mg/day, median duration of follow-up 147 days	Improvement in fatigue, pain, brain fog or dyspnoea at least 1 month after starting treatment using study questionnaire	No information provided

FSS, Functional Status Scale; LDN, low-dose naltrexone; NAD, nicotinamide adenine dinucleotide; PEM, post-exertional malaise; SF-36, 36-Item Short Form Survey.

**Figure 2:**
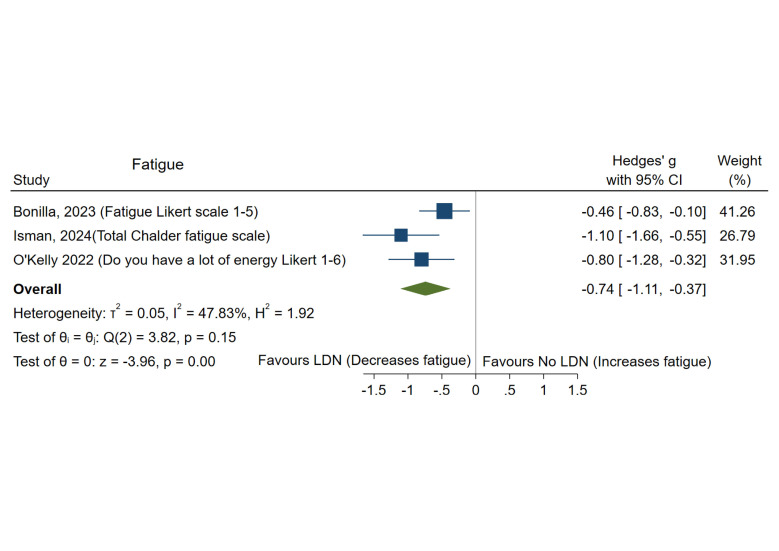
Forest plot of fatigue scores. LDN, low-dose naltrexone.

Forest plots (figures2^6^) summarise the effect sizes (Hedges’ g) from individual studies and the pooled effect sizes for each outcome. Scale directions were standardised so that negative values consistently favoured LDN. Figure 7 presents all effect sizes in a single combined plot. A detailed summary of outcomes is provided in (online supplemental 2).

**Figure 3:**
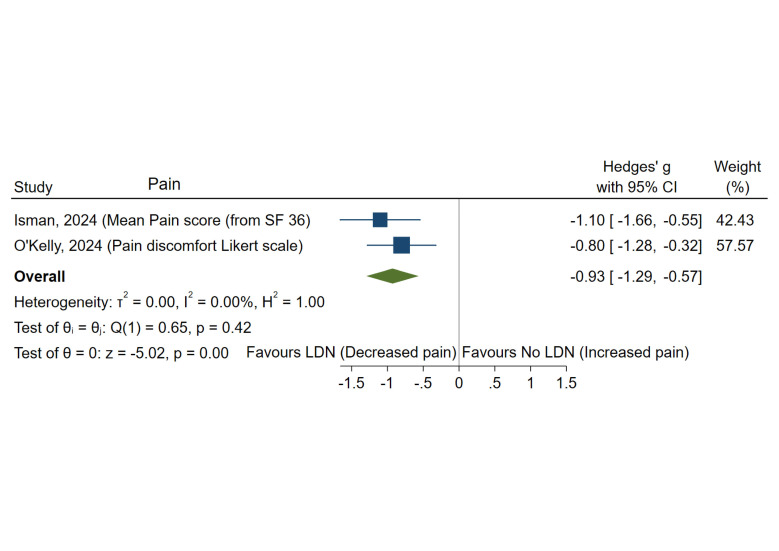
Forest plot of pain scores. LDN, low-dose naltrexone. SF-36, 36-Item Short Form Survey.

**Figure 4:**
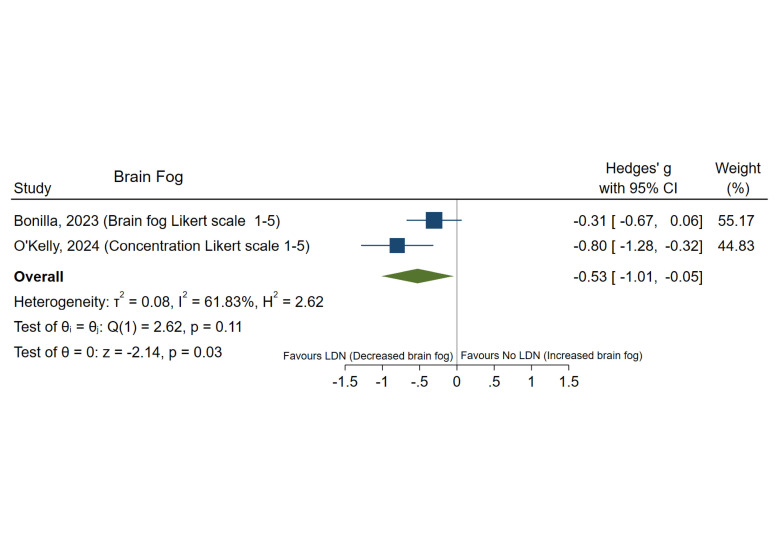
Forest plot of brain fog scores. LDN, low-dose naltrexone.

**Figure 5:**
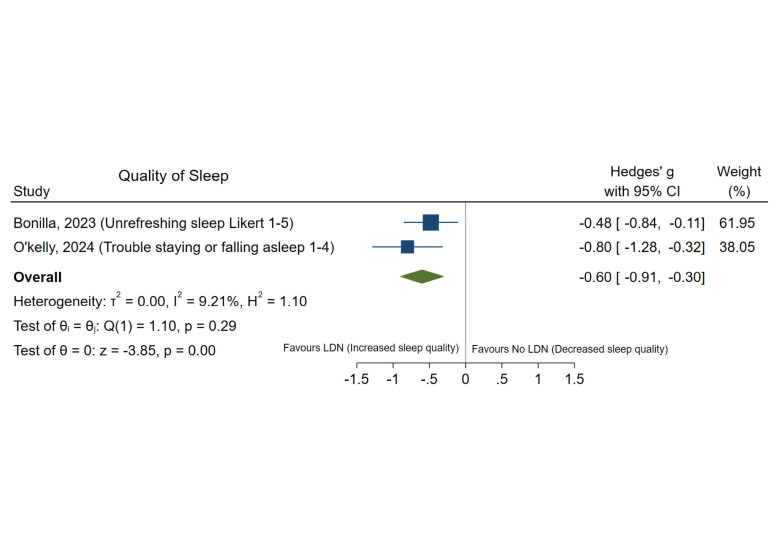
Forest plot of quality of sleep. LDN, low-dose naltrexone.

**Figure 6:**
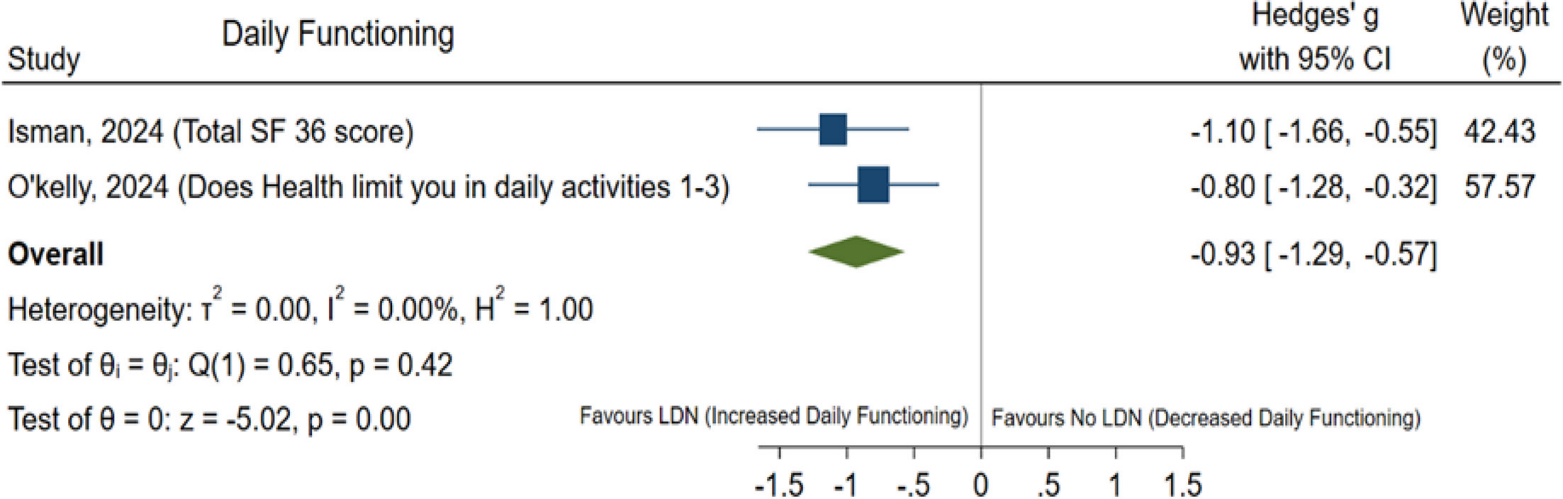
Forest plot of daily functioning scores. LDN, low-dose naltrexone. SF-36, 36-Item Short Form Survey.

**Figure 7:**
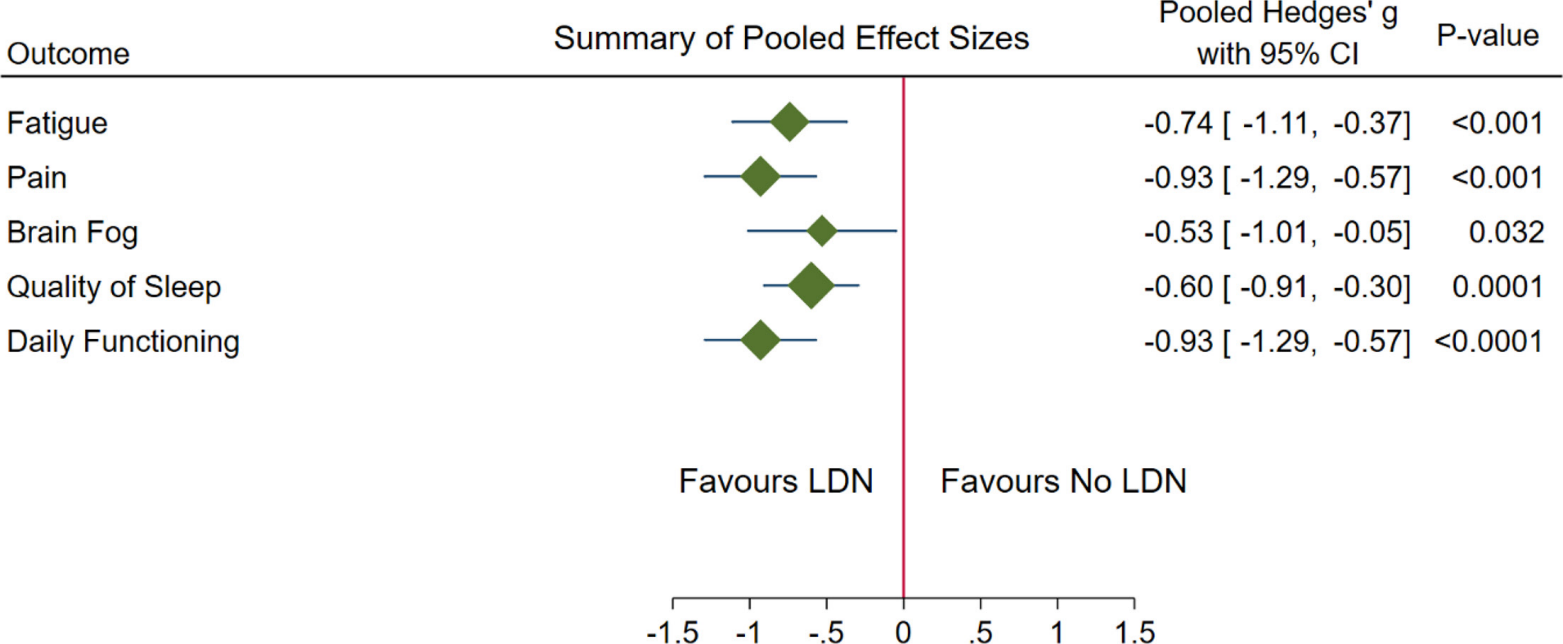
Summary of pooled effect sizes for all outcomes. LDN, low-dose naltrexone.

### Fatigue

Three studies reported fatigue outcomes, which were pooled.^21^,^23^ The meta-analysis showed a moderate effect size favouring LDN for reducing fatigue (Hedges’ g=−0.74; 95% CI −1.11 to −0.37; p<0.001; I^2^=47.7%) (figure 2). Two additional fatigue-related outcomes also favoured LDN: post exertional malaise from^21^; Hedges’ g=−0.48; 95% CI −0.84 to −0.11; p=0.010) and the 36-Item Short Form Survey (SF-36) energy/fatigue subscale^22^; Hedges’ g=−1.10; 95% CI −1.66 to −0.55; p<0.0001) (online supplemental 2). Tamariz *et al* reported fatigue improvement as a proportion of participants (13/24, 54.2%) and was not included in the meta-analysis, as it could not be converted to an SMD^24^ (online supplemental 2).

### Pain

Two studies reported pain outcomes, which were pooled.^22 23^ The meta-analysis showed a large effect size favouring LDN for reducing pain (Hedges’ g=−0.93; 95% CI −1.29 to −0.57; p<0.001) with no heterogeneity (I^2^=0%) (figure 3). Tamariz *et al* also reported improvement in pain as a proportion of participants (12/24, 50%) and was not included in the meta-analysis^24^ (see above; online supplemental 2).

### Brain fog

Two studies reported brain fog outcomes, which were pooled.^21 23^ The meta-analysis showed a moderate effect size favouring LDN for reducing brain fog (Hedges’ g=−0.53; 95% CI −1.01 to −0.05; p=0.03) (figure 4) with moderate heterogeneity (I^2^=61.8%). Tamariz *et al* also reported improvement in brain fog as a proportion of participants (4/24, 16.7%) and was not included in the meta-analysis^24^ (see above; online supplemental 2).

### Quality of sleep

Two studies reported sleep quality outcomes, which were pooled.^21 23^ The meta-analysis showed a moderate effect size favouring LDN for improving sleep quality (Hedges’ g=−0.60; 95% CI −0.91 to −0.30; p=0.0001) with low heterogeneity (I^2^=9.2%). Additionally, Bonilla *et al* reported a small effect on reducing an abnormal sleep pattern favouring LDN (Hedges’ g=−0.45; 95% CI −0.81 to −0.08; p=0.016)^21^ (online supplemental 2).

### Daily functioning

Two studies reported daily functioning outcomes, which were pooled.^22 23^ The meta-analysis showed a large effect favouring LDN for improving daily functioning (Hedges’ g=−0.93; 95% CI −1.29 to −0.57; p<0.0001) with no heterogeneity (I^2^=0.0 %) (figure 6).

### Summary of pooled effects

Figure 7 presents the pooled effect sizes for all five outcomes. LDN demonstrated moderate to large effects across fatigue, pain, brain fog, sleep quality and daily functioning.

## Risk of bias

Table 2 summarises the risk of bias for the included studies using the NOS.^17^ The maximum points for the selection domain were reduced by two to account for the non-applicability of ‘selection of the non-exposed cohort’ and the ‘outcome of interest was not present at the start of the study’. The comparability domain was reduced by one point due to the inapplicability of ‘the study controls for the most important factor’. Consequently, the maximum overall quality score was 6 points. All four studies scored either 4 or 5 points, indicating relatively low methodological concerns within the constraints of uncontrolled pre–post study designs.

**Table 2:** Risk of bias of included studies by Newcastle-Ottawa Scale

Assessment domains	Selection (Max 2 points)				Comparability (Max 1 point)	Outcome (Max 3 points)			Overall quality scores (max 6 points)	Risk of bias level
	Representativeness of the sample	Selection of non-exposed cohort	Ascertainment of exposure	Outcome of interest was not present at the start of study	Comparability based on design and analysis	Assessment of the outcome	Outcome follow-up	Adequacy of follow-up		
Bonilla 2023	1	N/A	1	N/A	1	0	1	1	5	Low
Isman 2024	1	N/A	1	N/A	1	0	1	0	4	Low
O’Kelly 2022	1	N/A	1	N/A	0	0	1	1	4	Low
Tamariz 2024	1	N/A	1	N/A	1	0	1	1	5	Low

### Registered trials

We identified four ongoing registered trials investigating LDN for long covid (table 3). The first is a phase 2 double-blind placebo-controlled RCT in British Columbia, Canada, evaluating titrated doses of LDN for post-COVID-19 fatigue syndrome over 16 weeks, with a target enrolment of 160 participants and an expected completion date of December 2024.^25 26^ The second is a phase-1 trial in Australia testing titrated dose of LDN for post-COVID-19 condition over 12 weeks, with a planned recruitment of 56 participants.^27^ The third, also led by the same Australian team, is assessing LDN for both ME/CFS and long covid.^28^ The fourth study is a randomised factorial group double-blinded placebo-controlled trial that is evaluating the effect and dosage of pyridostigmine (mestinon) and LDN in people with ME/CFS, long covid and orthostatic intolerance.^29^

**Table 3:** Summary of registered trials (n=4)

Registration ID	Location	Intervention and dose	Sample size	Treatment duration	Primary outcome
NCT05430152^25 26^	Canada	LDN as a compounded capsule starting at 1 mg/day and increasing up to 4.5 mg/day (by week 4)	n=160	16 weeks	Change in fatigue intensity by 4.7 points over using the Fatigue Severity Scale
ACTRN 12623001042639^27^ NALCOVID study	Australia	LDN 3–6 mg/day	n=56	12 weeks	Detectable change in symptom presentation and severity measured by DePaul Symptom Questionnaire
ACTRN 12624001162505^28^ TreatMELC study	Australia	LDN start at 1.5 mg/day and increase by 1.5 mg/day weekly until their maximum dose is reached (target 4–6 mg/day)	n=56	12 weeks	Change in the transient receptor potential cation channel subfamily M member 3 (TRPM3) function
NCT06366724^29^ LIFT: Life Improvement Trial	USA	LDN dosage timeline: Weeks 0–2: 1.5 mg/day Weeks 2–4: 3.0 mg/day Weeks 5–13: 4.5 mg/day	n=160	13 weeks	Functional capacity measured by Functional Capacity Questionnaire with 55 questions (FUNCAP55)

LDN, low-dose naltrexone.

## Discussion

### Principal findings

We found no RCTs, but four observational studies reported outcomes of LDN for long covid. Across these studies, pre–post analyses suggested improvements in fatigue, brain fog, pain, sleep quality and daily functioning, with SMDs ranging from 0.53 for brain fog to 0.93 for daily functioning. However, given the small number of included studies and their uncontrolled pre–post design, these estimates should be interpreted with caution and are primarily hypothesis-generating. No serious adverse events were reported. All four studies were non-randomised uncontrolled pre–post studies. Although most were assessed as having relatively low methodological concerns using the adapted NOS, the overall certainty of evidence is likely low when considered against the Grading of Recommendations Assessment, Development, and Evaluation (GRADE) tool key domains, including risk of bias, inconsistency, indirectness, and imprecision. A formal GRADE assessment was not conducted.

### Strengths and limitations

The strengths of our review include a comprehensive search of multiple databases for both published and unpublished studies and a structured risk of bias assessment to identify potential sources of bias. However, the uncontrolled design of included studies precludes causal inference. These results have several limitations. In particular the observed improvements could reflect the natural history of the illness, regression to the mean or placebo effects, as all outcomes were self-reported. Another limitation was the heterogeneity of outcome measures used across studies, which complicate pooling and interpretation of results.

### Interpretation and comparison with other evidence

The effect sizes observed in this review are comparable to or greater than those reported for interventions trialled in ME/CFS and fibromyalgia, conditions that share symptomatic and mechanistic overlap with long covid.^9 10^ For example, pharmacological agents used for chronic fatigue and chronic pain often achieve only small improvements in symptom scores. The magnitude of change associated with LDN, if replicated in controlled trials, could therefore represent a clinically meaningful advance. Mechanistically, LDN may reduce neuroinflammation through modulation of microglial activation and antagonism of toll-like receptor 4, and may restore TRPM3 ion channel function, which is impaired in both long covid and ME/CFS.^7 30^ However, this is unlikely to be the sole pathway. LDN has also been suggested to exert broader immunomodulatory and neuroinflammatory effects, although the precise mechanisms remain under investigation.^9 11 12^

### Implications for current practice and future research

LDN has been proposed for ME/CFS and other chronic inflammatory conditions, with case reports and series suggesting possible benefit, but controlled trials remain lacking for long covid. Despite this limited evidence, two recent clinical guides on infection-associated chronic illness management that included long covid recommended LDN as a potential treatment for fatigue, pain and post-exertional malaise.^30 31^ ^32^ Its oral administration and established safety profile make it an attractive candidate for repurposing if effectiveness is confirmed.

However, accessibility is currently limited because LDN must be compounded into low dose preparations and many clinicians are reluctant to prescribe it off-label. If robust evidence of effectiveness emerges, these barriers could be addressed, making LDN a low-cost treatment option that reduces financial burden for patients and expands access to care. In that scenario, LDN could provide a more feasible and equitable option than higher-cost alternatives such as antivirals or biologics, particularly in resource-limited settings where treatment choices for long covid are scarce.

We identified four ongoing trials testing LDN in long covid, two of which are randomised and controlled. These studies will be crucial in confirming efficacy, clarifying optimal dosing and titration schedules and establishing treatment duration. Future trials should incorporate validated patient-reported outcomes as well as objective measures of fatigue, cognitive function and physical activity. Stratification by symptom phenotype and comorbidities will also be important to identify subgroups most likely to benefit.

Together, these considerations underscore the need for well-designed, high-quality trials to establish whether LDN can be safely and effectively integrated into long covid management.

## Conclusion

Preliminary observational evidence suggests LDN may improve fatigue, brain fog, pain, sleep quality and daily functioning in patients with long covid. However, the evidence summarised here is of low certainty. A well-powered RCT is needed to establish efficacy, determine optimal use and confirm safety in this population. Given the global burden of long covid and the accessibility of LDN, high-quality research is warranted to clarify its role in clinical practice.

## Supplementary material

10.1136/bmjopen-2025-111253 online supplemental file 1

10.1136/bmjopen-2025-111253 online supplemental file 2

## Data Availability

All data relevant to the study are included in the article or uploaded as supplementary information.
