# Supplementary material for: Effect of low-dose naltrexone for long COVID: a systematic review and meta-analysis
Source: BMJ Open. 2026 Jul 16;16(7):e111253. doi: 10.1136/bmjopen-2025-111253 (PMC13384149; doi:10.1136/bmjopen-2025-111253)
Supplement: Supplementary file 3 [file bmjopen-2025-111253.reviewer_comments.pdf]

PEER REVIEW HISTORY

BMJ Open publishes all reviews undertaken for accepted manuscripts. Reviewers are asked to complete a checklist review form and are provided with free text boxes to elaborate on their assessment. These free text comments are reproduced below.

ARTICLE DETAILS

Title (Provisional)

Effect of low dose naltrexone for long covid: a systematic review and meta-analysis

Authors

Byambasuren, Oyungerel; Atkins, Tiffany; Baptista, Shaira; Glasziou, Paul; Chakraborty, Samantha

---

VERSION 1 - REVIEW

**Reviewer**	**1**
**Name**	**Dack, Kyle**
**Affiliation**	**University of Bristol, MRC Integrative Epidemiology Unit**
**Date**	**11-Nov-2025**
**COI**	**None**

Statistical review summary:

While this systematic review identified only a handful of studies, it makes good use of them to generate pooled estimates. The manuscript is clearly written and for the most part adheres to PRISMA reporting guidelines, and statistical best practices.

However, there are two significant omissions. First, a certainty of evidence assessment was not performed (i.e. GRADE) which is important to ensure readers interpret the findings with an appropriate level of confidence. Especially because the meta-analyses are quite small. Second, a risk of bias tool was used which tends to have poor sensitivity and is not recommended in handbooks such as Cochrane – more up to date tools are available. If the authors could revise to incorporate these two items, this would allow readers to have more confidence in the conclusions.

Abstract:

1. The design and study title should include “meta analysis” so that readers know it includes a quantitative synthesis.

2. To align with PRISMA please state the quantitative methods used, e.g. the meta-analysis type.

Methods:

3. Paragraph 3 – the search strategy is thorough and clearly reported, but would benefit from a subheading to signpost it, and the search date being reported.

4. RoB: My primary concern regarding the review is that observational studies were evaluated using the NOS, but this is known for having poor sensitivity to distinguish between high and low quality studies (as demonstrated in table 2), and is not recommended in current guides such as Cochrane. The tool recommended in the Cochrane handbook is ROBINS-I, which is more transparent, and focuses on a wider variety of potential biases. I recommend applying it so that the results can be considered as reliable as possible: https://methods.cochrane.org/bias/risk-bias-non-randomized-studies-interventions

5. Study outcomes: Outcomes were listed briefly as part of the eligibility criteria. However, PRISMA item 10a requires more detailed elaboration – the definition of outcomes, any limits on timeframe (e.g. followup), any specific criteria used to include or exclude each outcome (e.g. did it need to be using an externally validated tool), and how results from different scales were made compatible.

6. "We calculated Hedges' g effect sizes for pre–post differences." How was this calculated? I suggest elaborating to ensure replicability – e.g. any specific software, functions, which were used.

7. Certainty of evidence: This item is marked as NA in the PRISMA checklist but is a core component of the methodology, allowing readers to understand how confidently each result can be interpreted. In the manuscript there are references to certainty of evidence, but no formal results are reported. GRADE is the most common approach, which I recommend applying and reporting with each result: https://www.cochrane.org/authors/handbooks-and-manuals/handbook/current/chapter-14#section-14-2

Results

8. Figure 1. On the right hand side, 141 studies are identified through citation searching, but the "records removed before screening" box is missing meaning readers cannot know why these were not sought for retrieval.

9. Meta-analysis results: The results are clearly reported, including a helpful comment on the effect size and heterogeneity. However, this is followed by a sentence reporting another study finding, e.g. in the "Pain" subsection, after reporting the meta-analysis, a result from Tamariz 2024 is reported. It is not reported why this result is presented separately and not part of the meta-analysis. Other sections have similar additions, I suggest explaining in the text why these were not suitable for meta-analysis – for example, by stating the specific outcome tools used.

**Reviewer**	**2**
**Name**	**Tamariz, Leonardo**
**Affiliation**	**University of Miami Leonard M Miller School of Medicine, Department of Public Health Sciences**
**Date**	**29-Dec-2025**
**COI**	**Yes, my study is included in the review.**

The study summarizes the evidence found about LDN and Post COVID.

The following are concerns:

1. Provide a rationale for only using means as effect measures instead of relative risk. Several of the included studies reported categorical outcomes (i.e improvement in symptoms). Using relative risk might increase the number of studies in the forest plot.

2. The main concern is the small number of studies.

3. Only one of the forest plots has a I2 of >50 and the authors still used random effects models, please provide rationale.

4. The outcomes of included studies are not well described, this is important because they are all different as stated by the authors.

5. Publication bias is not reported.

6. It is unclear what the comparison group is in the included studies.

7. Provide a rationale for not including Gertner. Cureus. 2024.

8. Was there any disagreements between authors in the risk of bias and inclusion of studies.

9. Id like to see more discussion about how it works. Is the TRPM3 the only mechanism

10. The authors should comment Sep. BMC Infectious disease.

VERSION 1 - AUTHOR RESPONSE

Reviewer: 1
Dr. Kyle Dack, University of Bristol
Comments to the Author:
Statistical review summary:
While this systematic review identified only a handful of studies, it makes good use of them to generate pooled estimates. The manuscript is clearly written and for the most part adheres to PRISMA reporting guidelines, and statistical best practices.

However, there are two significant omissions. First, a certainty of evidence assessment was

not performed (i.e. GRADE) which is important to ensure readers interpret the findings with an appropriate level of confidence. Especially because the meta-analyses are quite small. Second, a risk of bias tool was used which tends to have poor sensitivity and is not recommended in handbooks such as Cochrane – more up to date tools are available. If the authors could revise to incorporate these two items, this would allow readers to have more confidence in the conclusions.

Abstract:
1. The design and study title should include "meta analysis" so that readers know it includes a quantitative synthesis.
- We have now added meta-analysis to the title.
2. To align with PRISMA please state the quantitative methods used, e.g. the meta-analysis type.
- We have clarified in the Abstract that meta-analyses were conducted using random-effects models.

Methods:
3. Paragraph 3 – the search strategy is thorough and clearly reported, but would benefit from a subheading to signpost it, and the search date being reported.
- We have added a subheading ("Search strategy") in the Methods section (page 4) and clarified the search date. We also updated the search on 5 May 2026; no additional includable studies were identified, although one further registered trial was added.

4. RoB: My primary concern regarding the review is that observational studies were evaluated using the NOS, but this is known for having poor sensitivity to distinguish between high and low quality studies (as demonstrated in table 2), and is not recommended in current guides such as Cochrane. The tool recommended in the Cochrane handbook is ROBINS-I, which is more transparent, and focuses on a wider variety of potential biases. I recommend applying it so that the results can be considered as reliable as possible:
https://methods.cochrane.org/bias/risk-bias-non-randomized-studies-interventions
- We agree that ROBINS-I is the preferred Cochrane tool for non-randomised intervention studies. However, because all included studies were small uncontrolled before–after studies without comparator groups, several ROBINS-I domains could not be meaningfully applied and would inherently be judged at critical risk by design. We therefore used the Newcastle–Ottawa Scale as a pragmatic tool to provide a structured descriptive assessment across studies.
5. Study outcomes: Outcomes were listed briefly as part of the eligibility criteria. However, PRISMA item 10a requires more detailed elaboration – the definition of outcomes, any limits on timeframe (e.g. followup), any specific criteria used to include or exclude each outcome (e.g. did it need to be using an externally validated tool), and how results from different scales were made compatible.
- We have expanded on the Outcomes in the methods section (page 5) as follows:
The primary outcomes were fatigue and health-related quality of life. Secondary outcomes included cognitive symptoms or cognitive function, other long COVID–related symptoms, and adverse events (including gastrointestinal symptoms). Outcomes were included if they were measured using either validated patient-reported outcome measures or clearly defined symptom scales as reported by the original studies; no restrictions were placed on specific

instruments a priori due to expected heterogeneity in outcome measurement across early-stage studies.
Where studies reported outcomes using different validated scales measuring the same construct (e.g., fatigue or quality of life), results were standardised using standardised mean differences (SMDs) to enable comparability across instruments. Extracted outcomes reflected post-intervention timepoints reported in each study, with no restriction on follow-up duration due to variability in study design and reporting in the available literature. All available post-treatment assessments were included as reported.
No formal exclusion criteria were applied based on outcome measurement tool or follow-up duration, in order to capture the breadth of emerging evidence in this early field.
Given the heterogeneity and early-stage nature of the evidence base, outcome definitions and measurement instruments were accepted as reported in the primary studies, with synthesis undertaken descriptively and through standardisation where appropriate.
6. "We calculated Hedges' g effect sizes for pre–post differences." How was this calculated? I suggest elaborating to ensure replicability – e.g. any specific software, functions, which were used.
- We have expanded the Methods section to describe the calculation of Hedges' g in greater detail. Where effect sizes were not directly reported, they were derived from available summary statistics (including p-values for pre–post comparisons), using established statistical methods to obtain standardised mean differences, with small sample correction applied using Hedges' g. We have also clarified the use of the Campbell Collaboration effect size calculator (https://www.campbellcollaboration.org/calculator/d-t-p-equal or https://www.campbellcollaboration.org/calculator/d-t-p-unequal) and referenced the approach used to ensure transparency and reproducibility.
CHANGE: (Methods, page 6): Effect sizes for pre–post changes were calculated using Hedges' g (standardised mean difference with small-sample correction). Where effect sizes were not directly reported, they were derived from available summary statistics (e.g., p-values for within-group comparisons) using established methods, with approximations made where reporting was incomplete in the primary studies. Calculations were supported using the Campbell Collaboration effect size calculator and cross-checked for consistency, in line with guidance from the Cochrane Handbook for Systematic Reviews of Interventions.
7. Certainty of evidence: This item is marked as NA in the PRISMA checklist but is a core component of the methodology, allowing readers to understand how confidently each result can be interpreted. In the manuscript there are references to certainty of evidence, but no formal results are reported. GRADE is the most common approach, which I recommend applying and reporting with each result: https://www.cochrane.org/authors/handbooks-and-manuals/handbook/current/chapter-14#section-14-2
- This comment was addressed as part of the Editor's comment above.
Results
8. Figure 1. On the right hand side, 141 studies are identified through citation searching, but the "records removed before screening" box is missing meaning readers cannot know why these were not sought for retrieval.
- We followed the PRISMA 2020 flow diagram template. The 141 records identified through citation searching did not contribute additional unique studies after deduplication with records identified through database searching. As a result, no additional reports were taken forward for full-text retrieval or screening from this source.

9. Meta-analysis results: The results are clearly reported, including a helpful comment on the effect size and heterogeneity. However, this is followed by a sentence reporting another study finding, e.g. in the "Pain" subsection, after reporting the meta-analysis, a result from Tamariz 2024 is reported. It is not reported why this result is presented separately and not part of the meta-analysis. Other sections have similar additions, I suggest explaining in the text why these were not suitable for meta-analysis – for example, by stating the specific outcome tools used.
- We thank the reviewer for this helpful suggestion. Results that were not included in the meta-analysis were excluded due to differences in outcome reporting (e.g., non-continuous measures such as percentages, or insufficient data to calculate effect sizes). We have revised the Results section to explicitly state, for each instance, why these studies were not included in the meta-analysis. We have also clarified this approach in the Methods section (Data analysis) and provide all extracted outcomes in Supplement 2 for completeness.

CHANGE: We added the following sentences at each instance:
- Tamariz 2024 reported fatigue improvement as a proportion of participants (13/24, 54.2%) and was not included in the meta-analysis, as it could not be converted to a standardised mean difference (21).
- Tamariz 2024 also reported improvement in pain as a proportion of participants (12/24, 50%) and was not included in the meta-analysis (21) (see above; Supplement 2).
- Tamariz 2024 also reported improvement in brain fog as a proportion of participants (4/24, 16.7%) and was not included in the meta-analysis (21) (see above; Supplement 2).

Reviewer: 2
Dr. Leonardo Tamariz, University of Miami Leonard M Miller School of Medicine
Comments to the Author:
The study summarizes the evidence found about LDN and Post COVID.
The following are concerns:
1. Provide a rationale for only using means as effect measures instead of relative risk. Several of the included studies reported categorical outcomes (i.e improvement in symptoms). Using relative risk might increase the number of studies in the forest plot.
- We thank the reviewer for this important suggestion. While several included studies reported categorical symptom improvement, a relative risk–based synthesis was not feasible due to substantial heterogeneity in outcome definitions and reporting formats, as well as the lack of baseline comparator data in several studies. In particular, some studies reported only post-intervention proportions without pre-intervention values, preventing calculation of relative risk, while others reported symptom counts or continuous symptom scales. To enable a consistent synthesis across studies, we therefore standardised outcomes to continuous effect sizes (Hedges' g), which allowed inclusion of both categorical and continuous measures within a unified analytical framework. All study-level categorical outcomes are reported in Supplementary Table 2 for transparency.
2. The main concern is the small number of studies.
- We acknowledge that the small number of included studies is a key limitation of this review and reflects the current state of the evidence base for low-dose naltrexone in long COVID, rather than a limitation of the review process itself. Despite a comprehensive search strategy, only a small number of eligible studies meeting inclusion criteria were identified. We have highlighted this limitation more explicitly in the Discussion and have tempered interpretation

of the pooled estimates accordingly.
CHANGE: we added the following in the Discussion: Principal findings: “However, given the small number of included studies, pooled estimates should be interpreted with caution and are primarily hypothesis-generating.”
3. Only one of the forest plots has a I2 of >50 and the authors still used random effects models, please provide rationale.
- A random-effects model was selected a priori due to expected clinical and methodological heterogeneity across studies (e.g., differences in populations, intervention dose, and follow-up). Model choice was therefore not based on I^2 thresholds, which can be unreliable with small numbers of studies. Given the limited and heterogeneous evidence base, a random-effects approach was considered more appropriate.
CHANGE: we added the following to the Data Analysis section of the Methods (page 6): “A random-effects model was selected a priori to account for anticipated clinical and methodological heterogeneity across studies, irrespective of the magnitude of statistical heterogeneity (I^2).”
4. The outcomes of included studies are not well described, this is important because they are all different as stated by the authors.
- Outcome measures varied across studies, as noted in the manuscript. We reported descriptive details of the outcomes and measurement instruments in Table 1, with all numerical outcome data presented in the pooled analyses and Supplementary Table 2. We have also revised Table 1 to include the specific measurement tools used in each study for completeness.
5. Publication bias is not reported.
- Publication bias was not formally assessed due to the small number of included studies (<10), as recommended by methodological guidance, since funnel plots and related tests are unreliable with few studies.
6. It is unclear what the comparison group is in the included studies.
- We didn’t find any studies with comparison groups (ie, RCTs). All included studies were single-group before–after designs without a comparator arm. The analyses were therefore based on within-group pre–post changes rather than between-group comparisons.
7. Provide a rationale for not including Gertner. Cureus. 2024.
- The study by Gertner was not included as it did not meet our predefined population criteria. Specifically, it involved patients with acute COVID-19 rather than individuals with long COVID (≥12 weeks post-infection), which was required for inclusion in this review.
8. Was there any disagreements between authors in the risk of bias and inclusion of studies.
- Any minor differences in interpretation during study selection and risk-of-bias assessment were resolved through discussion between reviewers. The small number of included studies and clearly defined eligibility criteria contributed to a high level of agreement.
9. I’d like to see more discussion about how it works. Is the TRPM3 the only mechanism
- This review intended to summarise proposed mechanisms of low-dose naltrexone relevant to long COVID rather than provide an exhaustive mechanistic review. We have clarified that TRPM3 channel dysfunction is one proposed pathway supported by emerging evidence, but that it is unlikely to be the sole mechanism. Other potential mechanisms, including immunomodulatory and neuroinflammatory effects, may also contribute and remain under investigation.
CHANGE: We added the following sentence in the Discussion (page 14): “…However, this is unlikely to be the sole pathway. Low-dose naltrexone has also been suggested to exert

broader immunomodulatory and neuroinflammatory effects, although the precise mechanisms remain under investigation."

10. The authors should comment Sep. BMC Infectious disease.

- We would appreciate clarification of this comment regarding which specific article or aspect of the BMC Infectious Diseases issue is being referred to. We would be happy to address this once clarified.
